# Experience of obstetricians and gynecologists in the management of medicolegal cases in Egypt

**DOI:** 10.1186/s12905-022-02065-6

**Published:** 2022-12-24

**Authors:** Zahraa Khalifa Sobh, Esraa Hassan Ahmed Oraby, Saffa Abdelaziz Mohamed Abdelaziz

**Affiliations:** 1 Forensic Medicine and Clinical Toxicology Department, Faculty of Medicine, Alexandria University, Alexandria, Egypt; 2 Obstetrics and Gynecology Department, Faculty of Medicine, Alexandria University, Alexandria, Egypt

**Keywords:** Obstetrics and Gynecology, Medicolegal Cases, Malpractice, Hymen, Abortion; Egypt

## Abstract

**Background:**

Obstetrics and gynecology (OB/GYN) is concerned with both fetal and maternal wellbeing with doubled professional responsibilities. Also, OB/GYN specialists are engaged in management of medicolegal cases (MLC). This study had an in-depth look at handling of MLC by obstetricians and gynecologists in Egypt. Also, influence of religious and cultural norms on OB/GYN practice was investigated.

**Methods:**

A questionnaire was formulated in compliance with literature and modulated according to religious and cultural background.

**Results:**

Responses were received from practitioners in 19 governorates. 28% of respondents were accused of malpractice. 87.3% of participants denoted increasing rate of litigations over last years. The commonest mentioned error is intra-operative problems (60%), whereas the commonest harm is neonatal deaths (46.7%). The mean participants' awareness score regarding elements of malpractice was 1.49 ± 0.76 (maximum possible score is 4). 18.7% of respondents managed cases of sexual assaults and premarital sexual relations. Hymen repair is not justified by 53.3% of participants. Termination of pregnancy before 16 weeks without medical indication is unaccepted by 96.7% of participants. The mean participants' awareness score regarding precautions of therapeutic abortion was 3.34 ± 1.63 (maximum possible score is 5).

**Conclusions:**

OB/GYN specialists are at high risk of accusation with inadequate measures to guard against malpractice claims in Egypt. High controversy among respondents regarding management of cases with premarital sexual acts and termination of pregnancy was elucidated.

**Supplementary Information:**

The online version contains supplementary material available at 10.1186/s12905-022-02065-6.

## Introduction

Obstetric-gynecologic forensic medicine is the foundation of ethical clinical practice and medicolegal responsibilities in this field. As obstetrics and gynecology (OB/GYN) is concerned with both fetal and maternal wellbeing, professional responsibility is doubled in most clinical settings [1]. Worldwide, there is an alarming rise in litigations against physicians of different medical specialties. OB/GYN is considered among the high-risk professions with inevitable emergencies. Therefore, healthcare providers and maternity hospitals should carefully consider medicolegal aspects of medical practice [2].

Other than malpractice claims, obstetricians and gynecologists could be involved in critical medicolegal tasks. In Arabian cultures, including Egypt, chastity is linked to the honor of women and their families. Sexual activities are prohibited outside the marriage bond due to religious and social background. Therefore, the victims of sexual violence and females with premarital consensual sexual relationships usually seek OB/GYN specialists for help. Verification of hymen integrity, hymen repair, and prevention or even termination of pregnancy are frequently requested in these situations [3, 4].

Restoration of hymen integrity and abortion-related issues are controversial in Islamic jurisprudence that govern medical practice in most Arab countries. In addition, there is no consensus in medical ethics literature regarding the legality of these practices. Thus, OB/GYN specialists are frequently confronted by a wide range of medicolegal and ethical challenges in this context [3, 5].

Egypt is the largest Arabian country by population size and accounts for 34.3% of the Arabian population [6]. It is worth mentioning that OB/GYN in Egypt is considered a specialty carried out by the same physician [7]. Egyptian obstetricians and gynecologists manage cases in a nation with 102 million population that grows by 1.9% annually [8].

Medicolegal cases (MLC) are defined as medical cases with legal implications. Thus, in the field of OB/GYN, MLC referred to patients who raise malpractice claims in courts [2]. Also, cases with medicolegal significance such as those who asked for verification of hymen integrity or termination of pregnancy are considered MLC [3, 5]. To date, there are no published statistics considering the percentage of MLC in Egypt either in OB/GYN or other medical specialties. However, recent national studies pointed to an increase in MLC cases in Egypt [9, 10].

The experience of OB/GYN practitioners in dealing with medicolegal and ethically challenging situations is a lesson to learn [11]. Thus, this study takes an in-depth look at the attitude and practice of Egyptian obstetricians and gynecologists regarding the management of MLC. Also, the influence of religious and cultural norms on OB/GYN practice in Egypt was investigated.

## Subjects and methods

### Study design

This research is considered a cross-sectional descriptive study.

### Study participants

OB/GYN practitioners from different Egyptian governorates were invited to participate in the study. The questionnaire was available on medical pages on social media that were accessed by thousands of OB/GYN practitioners in Egypt. Also, OB/GYN practitioners in different Egyptian governorates were contacted and encouraged to participate in the survey and to distribute web-based questionnaires to their colleagues. The aim of the present work was elucidated. The invited participants were encouraged to share their experience in managing MLC in Egypt through a response to a self-reported questionnaire. The completion and submission of the questionnaire were considered implied consent for participation in this survey. Confidentiality of the respondents' data was maintained.

### Survey instrument and sampling

This web-based questionnaire was formulated following a thorough review of medicolegal and ethical literature in OB/GYN [1, 2, 12–14]. The questions were modulated to investigate the effect of the religious and cultural norms on the OB/GYN practice [3, 5, 15, 16]. Ethical approval was obtained from the Research Ethics Committee of the Faculty of Medicine, Alexandria University (IRB Number: 00012098, FWA Number: 00018699, Serial Protocol Number: 0305331).

This survey encompasses 19 questions that were assigned into 3 main sections. The first Sect. (6 questions) was concerned with the personal and professional data of the respondents. The questions of the second Sect. (7 questions) gathered information regarding malpractice claims against OB/GYN practitioners. The third section assessed the participants' management of cases with medicolegal significance (5 questions). The survey questions were designed in two forms: Single correct answer per question and multiple correct answers per question.

Before distributing the questionnaire, the content validity of the questions was judged by 3 senior experts: 2 forensic medicine consultants and an OB/GYN consultant. As regards face validity, 12 questions from Q7 to Q18 were examined using confirmatory factor analysis. The model divided them into two separate domains. It was revealed that questions from 7 to 13 were concerned with malpractice claims against OB/GYN practitioners (thus belonging to the second domain), and questions from 14 to 18 assessed participants’ management of cases with medicolegal significance (thus belonging to the third domain). Internal consistency reliability was tested using Cronbach’s alpha. For the second domain, Cronbach’s alpha was 0.861, and for the third domain, it was 0.792 [17].

The sample size was calculated using Epi Info software [18], considering confidence level 95%, power (80%), and prevalence of assurance practice among physicians 89% [19]. The calculated sample size = 150 participants.

Non-probability sampling method (convenience sampling) was used, and the responses were received from 150 OB/GYN practitioners in 19 governorates (out of 27 Egyptian governorates). A wide distribution of the questionnaire and receiving responses from 70% of governorates could compensate for the relatively limited participants’ number. Generally, limited participation could be attributed to the tendency for non-disclosure of work-related data or lack of interest in the research topic which is in agreement with Mansour et al. 2020 [20].

### Statistical methods

The data were analyzed using IBM SPSS software package version 20.0. (Armonk, NY: IBM Corp). Quantitative data were described using range (minimum and maximum), mean, standard deviation, and median. Kolmogorov–Smirnov test was used to verify the normality of data distribution. Chi-square, Monte Carlo, and Kruskal Wallis tests were implemented to compare different groups at a significance level 95%. Internal consistency reliability was tested using Cronbach’s alpha.

## Results

The present work included the responses of 150 OB/GYN practitioners from 19 Egyptian governorates. Two-thirds (66.1%) of the participants were between 30 and 40 years. Most of the respondents (85.4%) have practiced OB/GYN for less than 20 years old. A master's degree in OB/GYN was obtained by 79.3%. Specialists constituted 62% of the participants. More than half (58%) of the respondents are employed in Ministry of Health hospitals (Table 1).

**Table 1:** Descriptive Analysis of responses to 6 questions concerning personal and professional data of the participating OB/GYN practitioners (*n* = 150)

Q		Personal data	Number	Percentage
**1**	**Age (years)**^a^			
		< 30	19	12.7%
		30–40	99	66.1%
		40–50	16	10.6%
		> 50	16	10.6%
**2**	**Gender**^a^			
		Male	50	33.3%
		Female	100	66.7%
**3**	**Years of experience**^a^			
		< 10	76	50.7%
		10–20	52	34.7%
		> 20	22	14.7%
**4**	**Highest qualification**^a^			
		Bachelor	17	11.3%
		Master’s degree	119	79.3%
		Doctorate	14	9.3%
**5**	**Job level**^a^			
		Resident	27	18.0%
		Specialist	93	62.0%
		Consultant	30	20.0%
**6**	**Affiliated institute**^a^			
		University Hospital	38	25.3%
		Health ministry hospitals	87	58.0%
		Private sector	25	16.7%

^a^ Single correct answer per question

### Malpractice claims against OB/GYN practitioners (Table 2)

**Table 2:** Descriptive Analysis of responses to 7 questions discussing malpractice claims against OB/GYN practitioners (*n* = 150)

**Q**	**Questions discussing malpractice claims against OB/GYN practitioners**	No.	%
**7**	**Are you accused before of a malpractice claim?** ^**a**^		
	Yes	42	28.0%
	No	108	72.0%
**8**	**According to your observations, rate of malpractice claims in last years against OB/GYN** ^**a**^		
	Increasing	131	87.3%
	Stationary	17	11.3%
	Decreasing	2	1.3%
**9 **	**Cause(s) of increasing malpractice claims? (Optional according to response of Q2)** ^**b**^		
	Tendency of some patients to obtain compensation from physicians	91	60.7%
	Poor awareness of patients of the doctors' role in their management	65	43.3%
	Poor doctor-patient communication	49	32.7%
	Tendency of some physicians to achieve financial profit at expense of proper management	44	29.3%
	Decrease the efficiency of new generations of physicians	36	24.0%
	Increase awareness of patients regarding their rights	29	19.3%
**10**	**What are common OB/GYN practitioners errors?** ^**b**^		
	Intra-operative problems	90	60.0%
	Failure of prediction and management of possible complications.	62	41.3%
	Failure to obtain valid consent	53	35.3%
	Failure/delayed diagnosis	38	25.3%
	Extension of surgery beyond consent	28	18.7%
	Failure/delayed treatment	28	18.7%
	Operator error	23	15.3%
	Failed sterilization	22	14.7%
**11**	**What are common harms that make patients accuse OB/GYN practitioners?** ^**b**^		
	Neonatal deaths	70	46.7%
	Fetal injuries	69	46.0%
	Maternal deaths	67	44.7%
	Bowel injury	64	42.7%
	Bladder injury	46	30.7%
	Ureteric ligation/ injury	31	20.7%
	Secondary Infertility	24	16.0%
	Vascular injury	21	14.0%
	Incontinence	12	8.0%
**12**	**What are measures done in your institutes to protect OB/GYN practitioners against claim?** ^**b**^		
	Proper documentation	91	60.7%
	Training/ orientation of physicians regarding medicolegal issues	60	40.0%
	Regular meeting to discuss complicated cases and failure incidences	59	39.3%
	Communication/ counseling of patients	55	36.7%
	Increase educational standards of physicians	45	30.0%
	Implementation of quality standards for management of cases	32	21.3%
	Medical Insurance of physicians	29	19.3%
**13**	Physician is accused in malpractice claim if following element (s) is/are established: **		
	Injury or harm to the patient	81	54.0%
	A causal relation between medical error and patient harm	67	44.7%
	A medical error done by physicians	55	36.7%
	The professional duty of care of the patient	21	14.0%

^a^ Single correct answer per question

^b^ Multiple correct answers per question

The responses of participants to 7 questions discussing malpractice claims were analyzed. More than a quarter (28%) of respondents declared that they were previously accused of malpractice lawsuits before.

**Fig. 1:**
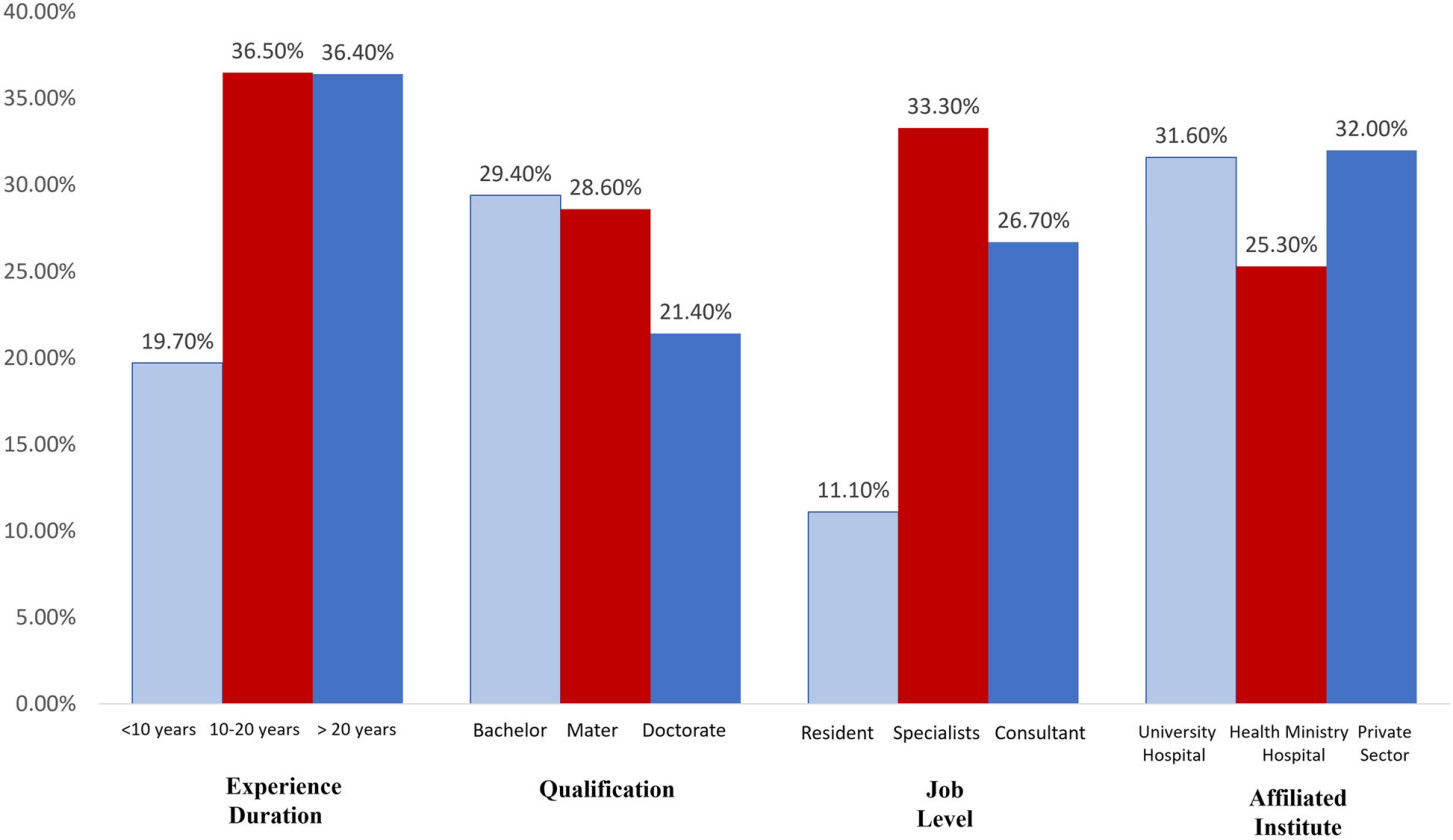
Personal and professional data of OB/GYN practitioners with approved malpractice claims (*n* = 42)

Figure (1) illustrates the personal and professional data of the participants with approved malpractice claims. Regarding the experience duration, the percentages of accused senior physicians with an experience duration of more than 10 years (> 36%) are higher than juniors (19.7%). Considering scientific degrees, those with bachelor's (29.4%) and master's (28.6%) degrees are more liable to be sued than more qualified physicians (21.4%). Regarding job level, specialists (33.3%) were accused more than consultants (26.7%) and resident doctors (11.1%). Approval of malpractice claims against physicians is not significantly affected by their experience duration (*p* = 0.074), qualification (0.892), job level (0.076), or affiliated institute (0.685).

Most of the participants (87.3%) declared that the medical liability litigations increased against OB/GYN practitioners over the last few years. Nearly two-thirds (60.7%) of the respondents attributed increasing claims to the tendency of some patients to obtain compensation from physicians. Whereas 43.3% and 32.7% denoted that patients' unawareness of the doctors' role in their management and inadequate doctor-patient communication respectively stand behind the malpractice litigations.

The commonest medical errors mentioned by the participating OB/GYN practitioners are intra-operative problems (60%), failure to predict and manage possible complications (41.3%), and failure to obtain valid consent (35.3%). The commonly reported harms are neonatal deaths (46.7%), fetal injuries (46%), and maternal deaths (44.7%).

The healthcare institutes take measures to protect healthcare providers against malpractice claims by ensuring proper documentation (60.7%), increasing the orientation of physicians regarding medicolegal issues (40%), conducting regular meetings to discuss complicated cases (39.3%), and counseling the patients (36.7%).

The participants were asked to identify the elements needed to approve malpractice claims against physicians that include:

- Injury or harm to the patient.
- A causal relation between medical error and patient harm.
- A medical error done by physicians.
- The professional duty of care of the patient.

Only 54% identified the patients’ harm as an element of malpractice, whereas the other 3 elements were less identified by respondents.

Awareness of participants regarding the four elements of malpractice was quantified. Each response from these 4 correct responses is scored by (1). Thus, the maximum possible score is (4). The participants' scores ranged from 1 to 4, with a mean of 1.49 ± 0.76 and a median of 1. Nearly two-thirds (64%) of participants identified only one correct answer (score 1). A quarter (25%) of participants identified two correct answers (score 2). Whereas 8% of participants identified three correct answers (score 3). Only 3% of participants identified all four correct answers (score 4).

There is no significant relationship between respondents' awareness and their experience duration, qualification, job level, and affiliated healthcare institutes with *p* values 0.603, 0.488, 0.707, and 0.283, respectively.

### Management of cases with medicolegal significance by OB/GYN practitioners (Table 3)

**Table 3:** Descriptive Analysis of responses to 5 questions discussing the management of cases with medicolegal significance by participating OB/GYN practitioners (*n* = 150)

**Q**	**Questions discussing management of cases of significant medicolegal importance **by **OB/GYN practitioners**	**No.**	**%**
**14 **	**Do you manage the cases with suspected sexual assault or premarital relations?** ^**a**^		
	Yes	28	18.7%
	No	122	81.3%
**15 **	**Following sexual assault or premarital relations, cases attend to…** ^**b**^		
	Assess the integrity of the hymen	115	76.7%
	Terminate illegal pregnancy	91	60.7%
	Ask for hymen repair	47	31.3%
	Need medicolegal reporting to denote that the hymen is intact	35	23.3%
**16 **	**In your opinion, hymen restoration surger y might be justified in…** ^**b**^		
	Non-justification of hymen repair under any circumstances	80	53.3%
	Following sexual assault (rape)	54	36.0%
	Accidental hymen injury	48	32.0%
	In girl <18 years with wellful sexual intercourse	3	2.0%
	In lady (> 18 years) has premarital sexual relations and want to marry	1	0.7%
**17 **	**In your opinion, is it justifiable to induce an abortion before 16 weeks of pregnancy upon patient request without medical indication?** ^**a**^		
	Yes	5	3.3%
	No	145	96.7%
**18 **	**The appropriate medicolegal precautions taken by the doctor performing therapeutic (legal) abortion include the following** ^**b**^		
	Written reports from two specialists (of the mother's illness) stating that the continuation of pregnancy endangers her life	109	72.7%
	The operation is performed only in a hospital	107	71.3%
	Written consent of the woman and her husband	101	67.3%
	Reports and details of all that was done are kept in the official files	98	65.3%
	The operation is performed only by OB/GYN practitioners	86	57.3%

^a^ Single correct answer per question

^b^ Multiple correct answers per question

The responses of participants to 5 questions discussing the management of medicolegal issues were analyzed. Cases with sexual assaults and premarital sexual acts were managed by 18.7% of the participating physicians.

**Fig. 2:**
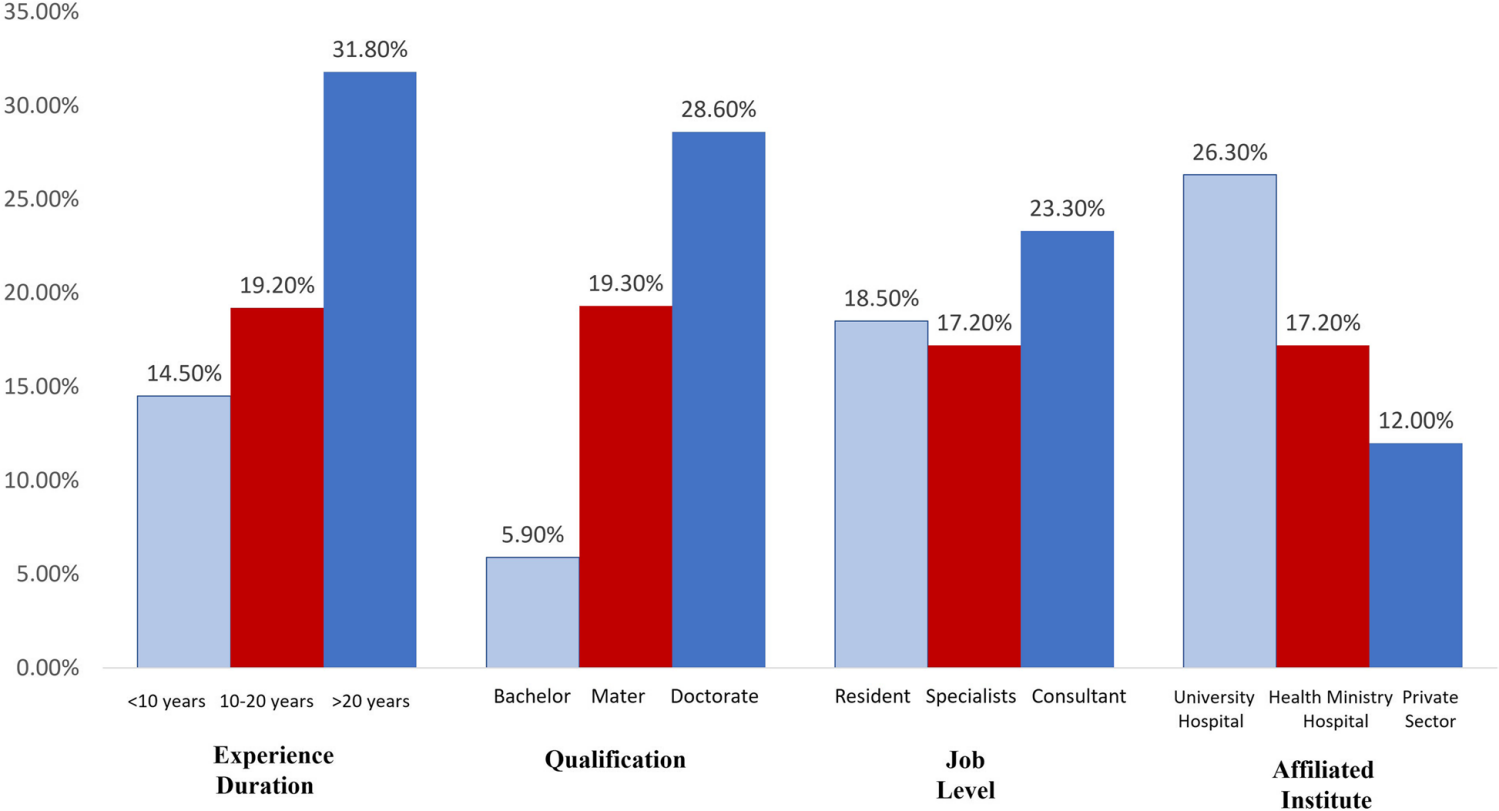
Personal and professional data of OB/GYN practitioners managing cases of sexual assault or premarital relations (*n* = 28)

Figure (2) shows the personal and professional data of the OB/GYN practitioners involved in managing cases of sexual assault or premarital relations. It was observed that these cases were managed by 31.8% of the most senior physicians, 28.6% of those who have a doctorate, and 23.3% of the consultants. Whereas junior practitioners, those with less qualifications and lower job levels, were less involved in managing cases of sexual assault or premarital relations. Physicians affiliated with university hospitals (26.3%) were more engaged in managing these cases than other physicians. The involvement of participants in managing cases of sexual assault or premarital relations is not significantly affected by their experience duration (*p* = 0.183), qualification (0.267), job level (0.755), or affiliated institute (0.315).

Assessment of the integrity of the hymen was identified by more than three-quarters (76.7%) of participants as the main concern of the females or their relatives. Whereas termination of extramarital pregnancy was mentioned by 60.7% of respondents. On the other hand, 31.3% and 23.3% of the participants stated that hymen repair and reporting of its integrity were the aims of the attending cases, respectively.

Hymen restoration surgery is not justified by more than half (53.3%) of the participants under any circumstances. Whereas more than a third (36%) of the responding physicians accepted hymen repair to the victims of sexual assault. Restoration of hymen integrity was justified by 32% of respondents if it was accidentally injured. On the other hand, only 2% and 0.7% agreed with hymen repair in minors and adults following consensual premarital sexual relationships, respectively. Regarding induction of abortion, only 3.3% justified termination of pregnancy before 16 weeks without medical indication.

The participants were asked to identify the appropriate medicolegal precautions taken by the doctor performing a therapeutic abortion that include:

- Written reports from two specialists (of the mother's illness) stating that the continuation of pregnancy endangers her life.
- The operation is performed only in a hospital.
- Written consent of the woman and her husband.
- Reports and details of all that was done are kept in the official files.
- The operation is performed only by OB/GYN practitioners.

Nearly three-quarters of respondents (72.7%) identified specialists’ reports stating that the continuation of pregnancy endangers a mother’s life as a precaution before the termination of pregnancy. The other 4 precautions were less identified by respondents.

Awareness of par ticipants regarding the appropriate medicolegal precautions of induction of therapeutic abortion was quantified. Each response from these five correct responses is scored by (1). Thus, the maximum possible score is (5). The participants' scores ranged from 1 to 5, with a mean of 3.34 ± 1.63 and a median of 4. Nearly a quarter (25.3) of the participants identified one correct answer (score 1), 6.7% of participants identified two correct answers (score 2), 14.7% of participants identified three correct answers (score 3), and 15.3% of participants identified four correct answers (score 4). Nevertheless, more than a third (38%) of participants identified all correct answers (score 5).

There is no significant relationship between respondents' awareness and their experience duration, qualification, job level, and affiliated healthcare institutes with *p* values 0.610, 0.367, 0.482, and 0.223, respectively.

## Discussion

OB/GYN practitioners are frequently confronted with critical and medicolegal situations. Investigations of malpractice-related issues are the first step in protecting physicians with high-risk specialties. Also, evaluation of MLC management guides future readjustment of potential defects in this context [1, 11]. Therefore, this study had an in-depth look at the handling of obstetricians and gynecologists of MLC in Egypt.

More than three-quarters of the respondents had a master's degree in OB/GYN. The highest percentage of the participants were aged between 30 and 40 years. The high participation of middle-aged physicians is attributed to their high accessibility to online pages through which the questionnaire was distributed. More senior doctors were often less skillful in accessing web-based sites than junior staff [20].

In the present work, nearly a third of respondents were previously accused of malpractice claims that denote that OB/GYN is a highly litigious specialty in Egypt. It is worth mentioning that Azab 2013 analyzed malpractice claims investigated by the Egyptian Medical Syndicate and found that obstetricians and gynecologists were the most sued physicians [21]. Similarly, Mashali et al. 2020 [22] denoted that OB/GYN practitioners were the most accused physicians of malpractice in Alexandria, the second biggest city in Egypt. In Saudi Arabia, AlDakhil 2016 declared more litigation against obstetricians and gynecologists in relation to other specialities [23].

In the United Kingdom, it was reported that 76% of OB/GYN practitioners faced a claim at least once [24]. Similarly, in USA, literature revealed that all obstetricians and gynecologists had been accused during their medical practice [25]. The restriction of these studies to senior practitioners with a long duration of medical practice explains the relatively high fraction of accused physicians in relation to the present study.

In the current research, the percentages of accused physicians with an experience more than 10 years are high compared with more junior staff. It seems that longer practice is associated with managing a larger number of cases with a subsequent higher probability of malpractice claims.

A higher scientific degree positively impacts the physicians' competency in managing complicated cases. Therefore, in the present work, the percentage of accused physicians is higher among those with a bachelor's degree than those who have master's and doctorate degrees. Regarding job level, the percentage of accused specialists was higher than that of consultants and resident doctors. The consultants' experience decreases the liability of their accusation of malpractice. Regarding resident doctors, they are unlikely to manage complicated cases without supervision, whereas the management of high-profile cases is the utmost duty of the specialists.

In this study, 87.3% of the respondents reported an increased rate of malpractice claims against OB/GYN practitioners in Egypt in the last few years. Nearly two-thirds of the participants attributed increasing claims to the false allegations of some patients to obtain compensation. Similarly, other Egyptian studies previously reported unjustified claims against physicians to obtain financial indemnity [21, 22]

In the present work, 43.3% and 32.7% of the participants mentioned that the limited awareness of patients regarding the doctors' role in their management and the inadequate doctor-patient communication cause increased malpractice litigations. It is worth mentioning that a considerable sector of Egyptian society encourages a large family size. The concept of high parity is prevailing among populations with low educational standards that explains their misperception and limited communication skills with healthcare providers [26].

Regarding medical errors, the study participants denoted that operative problem, unsuccessful management of complications, and failure to obtain valid consent are the commonest pitfalls associated with malpractice claims. Similar medical errors were previously reported by Gowda et al. 2016 [27] and Mashali et al. 2020 [22].

Considering the sequelae, the respondents pointed to neonatal deaths, fetal injuries, and maternal deaths as the commonest consequences of medical errors. Human suffering pushes the patients or relatives to take legal actions against healthcare providers and their institutes. AlDakhil 2016 mentioned similar maternal and fetal morbidities and mortalities in relation to medicolegal litigations against OB/GYN practitioners [23].

The participants' awareness of malpractice elements was unsatisfactory in the current study. The personal and professional characteristics didn't influence the knowledge regarding medical malpractice. It could be concluded that malpractice-related knowledge is neither included in postgraduate curricula nor acquired by clinical practice. Regarding healthcare institutes, serious defects were recorded in the measures taken to guard against malpractice claims. Ensuring proper medical documentation is mentioned by less than two-thirds of participants as the main protective action in Egyptian hospitals. Less identified protective measures include raising the medicolegal awareness of health care providers, discussing complicated cases in periodic meetings, enhancing the educational level of physicians, using quality standards in case management, and implementing medical insurance.

In oriental societies, including Egypt, the medical opinion regarding hymen integrity is associated with serious social and legal consequences [28]. Women might attend to obstetricians and gynecologists following sexual assaults or premarital sexual activities. Assessment of the hymen requires experience and must be done with caution. The inexperienced eye could perceive the normal variations such as hymenal notches and folds as defloration evidence [29, 30]. Therefore, in the current study, only 18.7% of participants handle cases of sexual assault or premarital relations. The management of these cases was mostly carried out by consultants and senior physicians with doctorate degrees.

Egyptian society valorizes premarital sexual purity due to religious and cultural beliefs [28, 30]. The current study pointed to the assessment of hymenal integrity and termination of pregnancy as the main concern of unmarried females. Also, restoration of hymen integrity could be requested if a medical assessment revealed hymenal defloration.

The practice of hymen restorative surgeries arouses religious and ethical controversies [5, 28, 31]. The current results reflect such a debate where more than half of the participants did not justify hymen restoration surgery under any circumstances. Other participants agreed on hymenal repair following sexual assault and accidental injuries. On the other hand, only a tiny fraction of the participants accept hymenal repair following consensual sexual acts.

Worldwide, medicolegal and ethical rules of termination of pregnancy are variable from total prohibition to unconditional permission. The international and national legislations are adapted to balance the right of the fetus to live and maternal autonomy to terminate pregnancy [32, 33]. In Egypt, the ethical clinical decision regarding the induction of abortion is regulated by the national medical practice law inspired by religious doctrines. Therapeutic abortion is permitted when the pregnancy endangers the mother's life or when fetal death is inevitable. Importantly, strict medicolegal precautions should be considered by the physician performing therapeutic abortion [3, 15]. In the current study, the awareness of most of the participants is satisfactory regarding the appropriate medicolegal precautions of induction of therapeutic abortion.

Some contemporary Islamic scholars permit the induction of abortion before 16 weeks of pregnancy in the absence of medical indication [3, 15]. However, only 3.3% of the current study participants justified induction of non-therapeutic abortion within the first 16 weeks of gestation. Refusal of the induction of abortion by the majority of the participants points to the sacredness of fetal life in Egyptian society.

### Recommendations

This study serves as a valuable reference that reflects the handling of MLC by Egyptian OB/GYN practitioners. In view of the current results, there is a necessity to enhance measures that protect physicians against malpractice claims, including providing training workshops and inclusion of medicolegal courses in OB/GYN curricula, improving communication with patients, and adopting medical insurance system. In addition, this study reflects controversies in managing cases with medicolegal significance in OB/GYN practice in Egypt. Subsequently, it is recommended to adopt a standardized policy that regulates the management of these cases. Also, it is recommended to carry out similar studies in different countries so that the comparability of the results with that of other nations could be possible.

### Limitations

The present work adopted a simple questionnaire to investigate OB/GYN practice in Egypt, the questions were either single correct answer per question or multiple correct answers per question. The relatively limited number of respondents and the absence of advanced question forms, such as Likert Scale questions, hindered more sophisticated statistical analyses; thus, there is a need to conduct a national study on a larger scale in Egypt following the enhancement of the survey instrument.

## Conclusions

This study denoted the high risk of accusation of OB/GYN practitioners in Egypt along with insufficient measures to protect healthcare providers. Also, the influence of Islamic jurisprudence and social norms on OB/GYN practice in Egypt was investigated, the results revealed a universal refusal of termination of the pregnancies in the early stages in absence of medical indication despite being accepted by some Islamic scholars. In addition, the study points to sexual purity as a major issue in Egyptian society and highlighted the debates regarding the conduction of hymen restoration following the loss of hymen integrity due to different circumstances.

## Supplementary Information

**Additional file 1: Table 1.** Relation between personal and professional data of participating OB/GYN practitioners and approval of malpractice claims against them (*n*= 42). **Table 2.** Relation between personal and professional data of participating OB/GYN practitioners and their awareness regarding the elements of malpractice claims (*n* = 150). **Table 3.** Relation between personal and professional data of participating OB/GYN practitioners and management of cases of sexual assault or premarital relations (*n*=28). **Table 4.** Relation between personal and professional data of participating OB/GYN practitioners and their awareness regarding appropriate medicolegal precautions of therapeutic abortion (*n* = 150).

## Data Availability

The datasets used during the current study are available from the corresponding author upon reasonable request.
