# Supplementary material for: Experience of obstetricians and gynecologists in the management of medicolegal cases in Egypt
Source: BMC Womens Health. 2022 Dec 24;22:544. doi: 10.1186/s12905-022-02065-6 (PMC9789585; doi:10.1186/s12905-022-02065-6)
Supplement: Supplementary file 1 — Additional file 1: Table 1. Relation between personal and professional data of participating OB/GYN practitioners and approval of malpractice claims against them (n= 42). Table 2. Relation between personal and professional data of participating OB/GYN practitioners and their awareness regarding the elements of malpractice claims (n = 150). Table 3. Relation between personal and professional data of participating OB/GYN practitioners and management of cases of sexual assault or premarital relations (n=28). Table 4. Relation between personal and professional data of participating OB/GYN practitioners and their awareness regarding appropriate medicolegal precautions of therapeutic abortion (n = 150). [file 12905_2022_2065_MOESM1_ESM.docx]

**Table 1** **Relation between personal and professional data of participating OB/GYN practitioners and approval of malpractice claims against them (n= 42).**

|  | **Participants with Approved Malpractice Claims** | | **All Participants** |
| --- | --- | --- | --- |
|  | **No.** | **% within each category** | **Total** |
| **Years of experience** |  |  |  |
| < 10 | 15 | 19.7 | **76** |
| 10 – 20 | 19 | 36.5 | **52** |
| > 20 | 8 | 36.4 | **22** |
| **χ^2^(p)** | 5.218 (0.074) | |  |
| **Highest qualification** |  |  |  |
| Bachelor | 5 | 29.4 | **17** |
| Master degree | 34 | 28.6 | **119** |
| Doctorate | 3 | 21.4 | **14** |
| **χ^2^(p)** | 0.311 (^MC^p=0.892) | |  |
| **Job level** |  |  |  |
| Resident | 3 | 11.1 | **27** |
| Specialist | 31 | 33.3 | **93** |
| Consultant | 8 | 26.7 | **30** |
| **χ^2^(p)** | 5.159 (0.076) | |  |
| **Affiliated institute** |  |  |  |
| University Hospital | 12 | 31.6 | **38** |
| Health ministry hospitals | 22 | 25.3 | **87** |
| Private sector | 8 | 32.0 | **25** |
| **χ^2^(p)** | 0.877 (0.685) | |  |

χ^2^: Chi square test MC: Monte Carlo

p: p value for comparing between the different categories

**Table 2** **Relation between personal and professional data of participating OB/GYN practitioners and their awareness regarding the elements of malpractice claims (n = 150).**

| **Personal data** | **Participants' awareness score regarding elements of malpractice** | | **H** | **P** |
| --- | --- | --- | --- | --- |
|  | **Mean ± SD.** | **Median** |  |  |
| **Years of experience** |  |  |  |  |
| < 10 | 1.53 ± 0.76 | 1.0 | 1.011 | 0.603 |
| 10 – 20 | 1.44 ± 0.78 | 1.0 |  |  |
| > 20 | 1.50 ± 0.74 | 1.0 |  |  |
| **Highest qualification** |  |  |  |  |
| Bachelor | 1.53 ± 0.62 | 1.0 | 1.434 | 0.488 |
| Master’s degree | 1.45 ± 0.72 | 1.0 |  |  |
| Doctorate | 1.79 ± 1.12 | 1.0 |  |  |
| **Job level** |  |  |  |  |
| Resident | 1.52 ± 0.64 | 1.0 | 0.693 | 0.707 |
| Specialist | 1.46 ± 0.75 | 1.0 |  |  |
| Consultant | 1.57 ± 0.90 | 1.0 |  |  |
| **Affiliated institute** |  |  |  |  |
| University Hospital | 1.71 ± 0.96 | 1.0 | 2.528 | 0.283 |
| Health ministry hospitals | 1.40 ± 0.62 | 1.0 |  |  |
| Private sector | 1.48 ± 0.82 | 1.0 |  |  |

H: H for Kruskal Wallis test

p: p value for comparing between the different categories

Maximum possible score = 4

**Table 3** **Relation between personal and professional data of participating OB/GYN practitioners and management of cases of sexual assault or premarital relations (n=28).**

|  | **Participants Managing Cases of Sexual Assaults/ Premarital Sexual Relation**  **(n= 28)** | | **All Participants**  **(n= 150)** |
| --- | --- | --- | --- |
|  | **No.** | **% within each category** | **Total** |
| **Years of experience** |  |  |  |
| < 10 | 11 | 14.5 | **76** |
| 10 – 20 | 10 | 19.2 | **52** |
| > 20 | 7 | 31.8 | **22** |
| **χ^2^(p)** | 3.397 (0.183) | |  |
| **Highest qualification** |  |  |  |
| Bachelor | 1 | 5.9 | **17** |
| Master degree | 23 | 19.3 | **119** |
| Doctorate | 4 | 28.6 | **14** |
| **χ^2^(p)** | 2.728 (^MC^p=0.267) | |  |
| **Job level** |  |  |  |
| Resident | 5 | 18.5 | **27** |
| Specialist | 16 | 17.2 | **93** |
| Consultant | 7 | 23.3 | **30** |
| **χ^2^(p)** | 0.562 (0.755) | |  |
| **Affiliated institute** |  |  |  |
| University Hospital | 10 | 26.3 | **38** |
| Health ministry hospitals | 15 | 17.2 | **87** |
| Private sector | 3 | 12.0 | **25** |
| **χ^2^(p)** | 2.313 (0.315) | |  |

χ^2^: Chi square test MC: Monte Carlo

p: p value for comparing between the different categories

**Table 4 Relation between** **personal and professional data of participating OB/GYN practitioners and their awareness regarding appropriate medicolegal precautions of therapeutic abortion (n = 150).**

| **Personal data** | **Participants' awareness score regarding precautions of therapeutic abortion** | | **H** | **P** |
| --- | --- | --- | --- | --- |
|  | **Mean ± SD.** | **Median** |  |  |
| **Years of experience** |  |  |  |  |
| < 10 | 3.43 ± 1.57 | 4.0 | 0.987 | 0.610 |
| 10 – 20 | 3.15 ± 1.68 | 3.0 |  |  |
| > 20 | 3.45 ± 1.74 | 4.0 |  |  |
| **Highest qualification** |  |  |  |  |
| Bachelor | 3.18 ± 1.81 | 3.0 | 2.004 | 0.367 |
| Master’s degree | 3.29 ± 1.63 | 4.0 |  |  |
| Doctorate | 3.93 ± 1.38 | 4.5 |  |  |
| **Job level** |  |  |  |  |
| Resident | 3.04 ± 1.63 | 3.0 | 1.459 | 0.482 |
| Specialist | 3.37 ± 1.65 | 4.0 |  |  |
| Consultant | 3.53 ± 1.59 | 4.0 |  |  |
| **Affiliated institute** |  |  |  |  |
| University Hospital | 3.71 ± 1.52 | 4.0 | 3.000 | 0.223 |
| Health ministry hospitals | 3.14 ± 1.69 | 3.0 |  |  |
| Private sector | 3.48 ± 1.53 | 4.0 |  |  |

H: H for Kruskal Wallis test

p: p value for comparing between the different categories
